# *Pantoea* Bacteriophage vB_PagS_Vid5: A Low-Temperature Siphovirus That Harbors a Cluster of Genes Involved in the Biosynthesis of Archaeosine

**DOI:** 10.3390/v10110583

**Published:** 2018-10-25

**Authors:** Eugenijus Šimoliūnas, Monika Šimoliūnienė, Laura Kaliniene, Aurelija Zajančkauskaitė, Martynas Skapas, Rolandas Meškys, Algirdas Kaupinis, Mindaugas Valius, Lidija Truncaitė

**Affiliations:** 1 Department of Molecular Microbiology and Biotechnology, Institute of Biochemistry, Life Sciences Centre, Vilnius University, Saulėtekio av. 7, LT-10257 Vilnius, Lithuania; monika.simoliuniene@gmail.com (M.Š.); laura.kaliniene@bchi.vu.lt (L.K.); aurelija.zajanckauskaite@bchi.vu.lt (A.Z.); rolandas.meskys@bchi.vu.lt (R.M.); 2 Center for Physical Sciences and Technology, Saulėtekio av. 3, LT-10257 Vilnius, Lithuania; martynas.skapas@gmail.com; 3 Proteomics Centre, Institute of Biochemistry, Life Sciences Centre, Vilnius University, Saulėtekio av. 7, LT-10257 Vilnius, Lithuania; algirdas.kaupinis@gf.vu.lt (A.K.); mindaugas.valius@bchi.vu.lt (M.V.)

**Keywords:** *Pantoea agglomerans*, vB_PagS_Vid5, LT bacteriophage, *Siphoviridae*, 7-deazaguanine

## Abstract

A novel low**-**temperature siphovirus, vB_PagS_Vid5 (Vid5), was isolated in Lithuania using *Pantoea agglomerans* isolate for the phage propagation. The 61,437 bp genome of Vid5 has a G–C content of 48.8% and contains 99 probable protein encoding genes and one gene for tRNA^Ser^. A comparative sequence analysis revealed that 46 out of 99 Vid5 open reading frames (ORFs) code for unique proteins that have no reliable identity to database entries. In total, 33 Vid5 ORFs were given a putative functional annotation, including those coding for the proteins responsible for virion morphogenesis, phage**-**host interactions, and DNA metabolism. In addition, a cluster of genes possibly involved in the biosynthesis of 7-deazaguanine derivatives was identified. Notably, one of these genes encodes a putative preQ_0_/preQ_1_ transporter, which has never been detected in bacteriophages to date. A proteomic analysis led to the experimental identification of 11 virion proteins, including nine that were predicted by bioinformatics approaches. Based on the phylogenetic analysis, Vid5 cannot be assigned to any genus currently recognized by ICTV, and may represent a new one within the family of *Siphoviridae*.

## 1. Introduction

*Pantoea* is a genus of highly diverse, yellow-pigmented, and rod-shaped Gram-negative bacteria of the family *Enterobacteriaceae*. Although members of this genus have been found to predominate in the phyllosphere of various plants, both as epiphytes and endophytes, *Pantoea* is being frequently isolated from many aquatic and terrestrial environments, and is also known to form host associations with insects, animals, and humans [1,2]. Despite this, only two bacteriophages with completely sequenced genomes, namely podoviruses LIMElight and LIMEzero [3], annotated as *Pantoea* phages, have been published in Genbank to date.

Nevertheless, it has been shown that a number of completely sequenced *Erwinia* bacteriophages are active on bacteria from the genus *Pantoea* [4,5,6,7,8,9]. It is not surprising, because *Pantoea* is closely related to *Tatumella* and *Erwinia*, the three forming a monophyletic group nested within the other enterobacterial genera, such as *Escherichia*, *Salmonella*, *Citrobacter*, *Enterobacter*, *Klebsiella,* and *Cronobacter* [1,10,11]. However, to our knowledge, all of the *Erwinia* bacteriophages characterized to date, with the exception of siphoviruses PhiEaH1 [12], PhiEaH2 [13], and KEY [14], have been assigned to the families *Myoviridae* and *Podoviridae*. This is rather unusual as, according to the literature, the *Siphoviridae* phages represent the majority of published bacterial viruses, and comprise the most abundant viral family within the order *Caudovirales* [15]. In addition, a virulent transducing siphovirus, phiOT8, which was isolated on *Serratia marcescens*, can also productively infect *Pantoea agglomerans* [16].

We present here the first report on the molecular characterization of siphovirus vB_PagS_Vid5 (below referred to by its shorter common laboratory name, Vid5, active on *Pantoea agglomerans*. Bacteriophage Vid5 shows a low-temperature plating profile, with an optimum temperature for plating of ~20 °C, and an ability to form plaques even at 4 °C. The phylogenetic analysis indicates that Vid5 has no close relatives within the family *Siphoviridae*. Moreover, a comparative genome sequence analysis suggests that Vid5 possesses a cluster of genes possibly involved in the biosynthesis of 7-deazaguanine derivatives. One of these genes codes for a putative preQ_0_/preQ_1_ transporter, which has never been detected in bacteriophages to date. Thus, the data presented here not only provide the information on *Pantoea*-infecting siphophages, but also offer novel insights into the diversity of the DNA modification systems used by bacteriophages.

## 2. Materials and Methods

### 2.1. Phages and Bacterial Strains

Bacteriophage Vid5 was originally isolated from the outwash of thicket shadbush berries collected in Lithuania using *Pantoea agglomerans* strain MMG as the host for phage propagation and phage growth experiments. The bacterial strains used in this study for host range determination are listed in Supplementary Table S1. For all of the phage experiments, the bacteria were cultivated in Luria–Bertani broth (LB) or LB agar. To identify isolated bacteria strains, A PCR-amplified 16S rRNA gene sequence analysis was performed. Universal primers woo1 and woo2 [17] were used for both the PCR amplification and the subsequent sequencing of the target 16S RNA gene.

### 2.2. Phage Techniques

Phage isolation was performed by using the enrichment of phages in the source material technique. Briefly, berries of thicket shadbush (50–100 g) were shaken for 1 h in 10 mL of LB. An aliquot (0.5 mL) of the outwash was transferred to the fresh LB medium (5 mL) and grown overnight at 25 °C, followed by a low-speed centrifugation at 5000 rpm for 15 min. The supernatant was then sequentially filtered through sterile 0.45 and 0.2 mm membrane filters, and was assayed for plaque forming units using the soft agar overlay method described by Adams [18], with minor modifications. Briefly, 0.1 mL of diluted phage suspension or clarified environmental samples were mixed with 0.5 mL of indicator cells (OD_600_-1). The mixture was then added to 2.5 mL of 0.5% (*w*/*v*) soft agar and was poured over the 1.2% LB agar plate as a uniform layer. The plates were incubated for 24–48 h at 20 °C before the enumeration of the plaques. Bacteriophage was purified by performing five consecutive transfers of phage from individual plaques to new bacterial cell lawns. Notably, as Vid5 propagated poorly in a liquid broth, the propagation of Vid5 was performed by the soft agar overlay method, as described previously [19], using *Pantoea agglomerans* strain MMG as a host. The phage purification was performed using a CsCl step gradient [20], as described previously [21].

### 2.3. Transmission Electron Microscopy

The CsCl density gradient-purified phage particles were diluted to approximately 10^11^ PFU/mL with distilled water, 5 mL of the sample was directly applied on the carbon-coated nickel grid (Agar Scientific, Essex, UK), the excess liquid was drained with filter paper before staining with two successive drops of 2% uranyl acetate (pH 4.5), dried, and examined in Tecnai G2 F20 X-TWIN transmission electron microscope (FEI, Hillsboro, OR, USA).

### 2.4. DNA Isolation and Restriction Analysis

The aliquots of phage suspension (10^11^–10^12^ PFU/mL) were subjected to phenol/chloroform extraction and ethanol precipitation, as described by Carlson and Miller [22]. The isolated phage DNA was subsequently used in restriction analysis, for PCR, or it was subjected to genome sequencing. The restriction digestion was performed with BamHI, Bsu15I, Csp6I, DraI, EcoRII, Eco32I, HhaI, MboI, MfeI, NdeI, and RsaI restriction endonucleases (Thermo Fisher Scientific, Vilnius, Lithuania), according to the supplier’s recommendations. The DNA fragments were separated by electrophoresis in a 0.8% agarose gel containing ethidium bromide. A restriction analysis was performed in triplicate to confirm the results.

### 2.5. Genome Sequencing and Analysis

The complete genome sequence of Vid5 was determined using Illumina DNA sequencing technology at BaseClear, in the Netherlands. Paired-end sequence reads were generated using the Illumina HiSeq2500 system (Illumina, San Diego, CA, USA). FASTQ sequence files were generated using the Illumina Casava pipeline version 1.8.3. An initial quality assessment was based on the data passing the Illumina Chastity filtering. The second quality assessment was based on the remaining reads using the FASTQC quality control tool version 0.10.0 (https://www.bioinformatics.babraham.ac.uk/projects/fastqc/).

The quality of the FASTQ sequences was enhanced by trimming off the low-quality bases using the “Trim sequences” option of the CLC Genomics Workbench version 8.5.1 (Qiagen, Hilden, Germany). The quality filtered sequence reads were puzzled into a number of contig sequences. The analysis was performed using the “De novo assembly” option of the CLC Genomics Workbench version 8.5.1. Misassemblies and nucleotide disagreement between the Illumina data and the contig sequences were corrected with Pilon version 1.11 (Broad Institute of MIT and Harvard, Cambridge, MA, USA). The contigs were linked and placed into scaffolds or supercontigs. The orientation, order, and distance between the contigs was estimated using the insert size between the paired-end and/or mate pair reads. The analysis was performed using the SSPACE Premium scaffolder version 2.3 (BaseClear B.V., Leiden, The Netherlands). The gapped regions within the scaffolds were (partially) closed in an automated manner using GapFiller version 1.10 (BaseClear, Leiden, the Netherlands). Thus, the reads were assembled into a single linear contig of 61,492 (4,515,811 reads; 6953 average coverage). The ends of the contig were confirmed using PCR, followed by Sanger sequencing reactions at Macrogen (Seoul, South Korea). A PCR fragment was obtained by the amplification of Vid5 wild-type DNA using Vid5_F 5′-GCGTACAACAATGTTCTAATCAG-3′ and Vid5_R 5′-GTCTTGCCAATGAAACGATAATG-3′ primers.

The open reading frames (ORFs) were predicted with Glimmer v3.02 (https://www.ncbi.nlm.nih.gov/genomes/MICROBES/glimmer_3.cgi) and Geneious v5.5.6. (http://www.geneious.com), using a minimum ORF size of 75 nt. The analysis of the genome sequence was performed using the Fasta-Protein, Fasta-Nucleotide, BLASTP, Transeq (http://www.ebi.ac.uk/Tools/st/emboss_transeq), and Clustal Omega (http://www.ebi.ac.uk/Tools/msa/clustalo), as well as HHPred, HHblits, HMMER, HHsenser, and Quick2D [23,24,25]. Also, tRNAscan-SE 1.21 (http://lowelab.ucsc.edu/tRNAscan-SE) was used to search for tRNAs. Phylogenetic analysis was conducted using MEGA version 5 [26] and Geneious v5.5.6. Whole genome alignment was performed using the mVista program in LAGAN mode [27] (http://genome.lbl.gov/vista). VIRFAM [28], (http://biodev.cea.fr/virfam) was used for the total proteome comparisons. The overall nucleotide sequence identity was calculated using PASC [29].

### 2.6. Analysis of Structural Proteins

An analysis of the Vid5 virion structural proteins was performed using a modified filter-aided sample preparation (FASP) protocol, followed by LC-MS/MS analysis, as described previously [19].

### 2.7. Nucleotide Sequence Accession Numbers

The complete genome sequence of the *Pantoea* bacteriophage, Vid5 was deposited in the EMBL nucleotide sequence database, under accession number MG948468. The accession numbers of the PCR-amplified 16S rRNA gene sequences of *Pantoea* isolates are as follows: *Pantoea agglomerans* strain ARC (MH158634), *Pantoea agglomerans* strain AUR (MH158652), *Pantoea agglomerans* strain BLS (MH158658), *Pantoea agglomerans* strain DDM (MH158728), *Pantoea agglomerans* strain MMG (MH158730), and *Pantoea agglomerans* strain SER (MH158734). The accession numbers of the phage genomes used in this study are as follows: Enterobacteria phage 9g (NC_024146), Enterobacteria phage CAjan (NC_028776), Enterobacteria phage JenK1 (NC_029021), Enterobacteria phage JenP1 (NC_029028), Enterobacteria phage JenP2 (NC_028997), *Escherichia* phage Greed (KX534337), *Escherichia* phage Seurat (KM236243), *Escherichia* phage slur01 (NC_028831), and *Escherichia* phage vB_Eco_SLUR25 (LT907986).

## 3. Results

### 3.1. Phage Morphology, Host Range, and Physiological Characteristics

**Figure 1:**
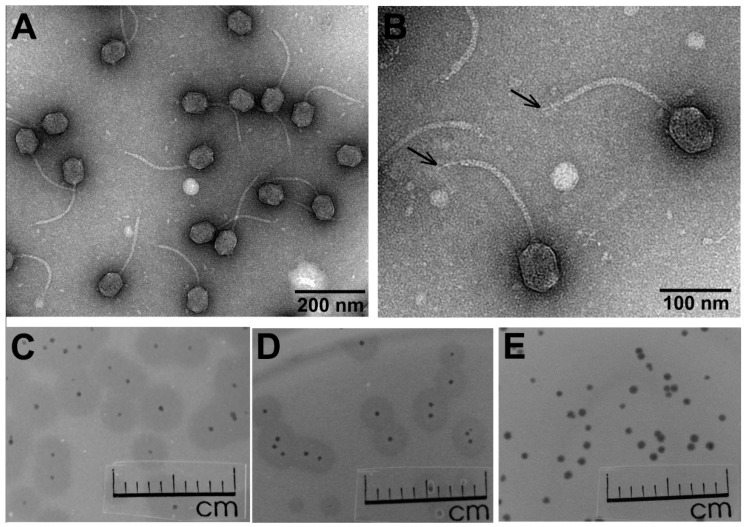
Electron micrographs of vB_PagS_Vid5 (Vid5) virions, and Vid5 plaque morphology. (**A**,**B**) CsCl-purified Vid5 phage particles. Black arrows indicate central tail fibers; plaques formed by Vid5 on a lawn of *Pantoea agglomerans* strain MMG after 96 h of incubation at 4 °C (**C**), 24 h at 15 °C (**D**), and 24 h at 22 °C (**E**).

Bacteriophage Vid5, together with its host *Pantoea agglomerans* strain MMG, was isolated from the outwash of a thicket of shadbush berries. Transmission electron microscopy observations of Vid5 (Figure 1A,B) revealed a particle that fits the B2 morphotype in Bradley’s classification [30,31]. Based on the morphological characteristics, phage Vid5 belongs to the family *Siphoviridae*, and is characterized by a slightly prolonged head (74.91 ± 3.03 nm [*n* = 48] in length and 53.73 ± 2.33 nm [*n* = 100] in width) and an apparently non-contractile tail (189.09 ± 12.86 nm in length [*n* = 40] and 9.01 ± 1.10 nm in width [*n* = 30]), with a single central tail fiber attached to a distal part of the tail. Notably, no side tail fibers were clearly visible using TEM.

In total, 26 bacterial strains (Supplementary Table S1) were used to explore the host range of Vid5. With the exception of *Pantoea agglomerans* strain MMG, the remaining five *Pantoea* sp. Isolates, as well as all of the tested strains of *Acinetobacter*, *Arthrobacter, Citrobacter*, *Enterobacter, Erwinia*, *Escherichia*, *Klebsiella*, *Salmonella*, and *Pseudomonas* spp. were found to be resistant to Vid5.

In order to determine the optimal conditions for phage propagation, the effect of temperature on the efficiency of plating (e.o.p.) was examined in the temperature range of 3–37 °C. The test revealed that Vid5 is a low-temperature virus—the phage forms plaques with a clear center surrounded by halo zone at 4–32 °C, and has an optimum temperature for plating about 22 °C (Figure 1C–E, Supplementary Figure S1). In addition, the morphology of the plaques formed by bacteriophage Vid5 depended on the cultivation temperature. After 24 h of incubation at 22 °C, the plaques were clear, up to 1 mm in diameter, surrounded by indistinct opaque halo zones (Figure 1E). After 24 h of incubation at 15 °C, the Vid5 plaques had a small clear center (up to 0.3 mm in diameter) surrounded by a clearly visible opaque halo zone up to 3 mm in diameter (Figure 1D). Moreover, the plaques with a clear center and 5 mm opaque halo zone were visible after 96 h of incubation at 4 °C (Figure 1C). According to the literature, the halo zones surrounding the phage plaques indicate the presence of bacterial exopolysaccharide (EPS)-degrading phage-encoded depolymerases [32]. Phage EPS-depolymerases act either as integral components of the virion particles or as free soluble enzymes. Within the halo zone, both phage particles and viable bacteria may be often found, and it has been suggested that halo formation is not only caused by the excess of EPS depolymerases produced inside the host during phage replication and released after cell lysis, but also by viral diffusion out of the primarily infected cell. Thus, the size of the halo is not only dependent on the activity of the phage-born depolymerase, but also on the number of phage produced in one single plaque [33,34]. Following these observations, there can be a number of reasons that, at different temperatures, bacteriophage Vid5 produces plaques of different morphologies. However, the most likely explanations are as follows: (i) upon infection at suboptimal temperatures (due to, e.g., delays in adsorption, low replication rate, etc.), less progeny phage particles and more free EPS-depolymerases are released after host lysis, and because these enzymes are substantially smaller than virions, they are able to diffuse further into bacterial lawns; (ii) the density of the bacterial lawn, because of the reduced growth rate, is lower at suboptimal temperatures, and hence the diffusion of phage or phage-associated EPS depolymerases out of the lysis zone is more effective, resulting in larger halo zones. Notably, bacteriophage Vid5 failed to reproduce after inoculation into liquid bacterial culture under investigated conditions, hence the adsorption and one-step growth experiments were not performed.

### 3.2. Vid5 Genomic and Proteomic Analysis

**Figure 2:**
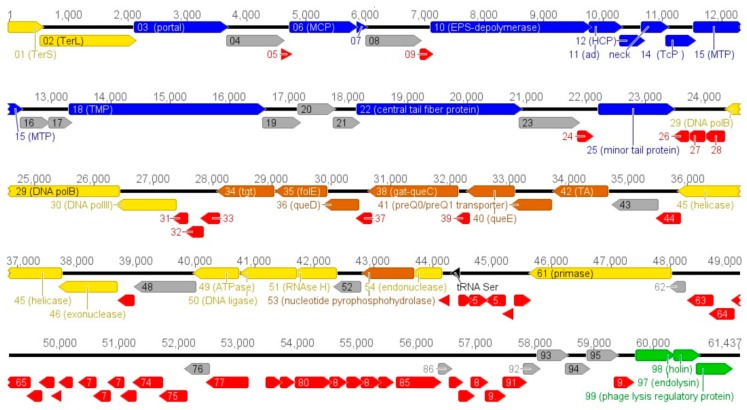
Functional genome map of bacteriophage Vid5. The coding capacity of the Vid5 genome is shown. The numbers indicate gene position in the genome, gene functions are assigned according to the characterized open reading frames (ORFs) in the NCBI database and HHpred analysis. The color code is as follows: yellow—DNA replication, recombination, repair, and packaging; brown—transcription, translation, and nucleotide metabolism; blue—structural proteins; purple—virion morphogenesis-related proteins; green—lysis, phage-host interaction; grey—ORFs of unknown function; red—Vid5 specific ORFs that encode unique proteins with no reliable identity to database entries, black—tRNA.

Phage Vid5 has a linear, double stranded DNA genome consisting of 61,437 bp with a G–C content of 48.8%, which differs from that (52–55%) observed for *Pantoea* spp. [1]. The results of the PCR and restriction-digestion analyses (data not shown) suggest that the Vid5 genome is a circularly permuted molecule. Similar to other dsDNA bacteriophages, the genome of Vid5 is close-packed—with an average ORF size of 599 bp, 96.5% of the genome is coding. The analysis of the genome sequence revealed that Vid5 has 99 probable protein-encoding genes and one gene for tRNA^Ser^ (Figure 2). Notably, an apparent symmetry in the distribution of the genes on the two DNA strands of phage Vid5 was observed. In total, 50 ORFs were predicted to be transcribed from the same DNA strand (including genes coding for structural and phage-host interaction proteins), while the other 49 ORFs (those involved in DNA replication, recombination, repair, and packaging, as well as transcription, translation, and nucleotide metabolism genes) were found on the opposite DNA strand (Figure 2).

A bioinformatics analysis revealed that 46 out of 99 Vid5 ORFs encode unique proteins that have no reliable identity (E-values > 0.001) to the database entries. In the case of Vid5 ORFs that encode proteins with matches to those in other sequenced genomes, the percentage of amino acid identity ranged from 28% to 77% and, in most cases (31 out of 53 Vid5 ORFs), from 43% to 58% (Supplementary Table S2). Among the Vid5 gene products with detectable homologs in other sequenced genomes, 49 were the most similar to proteins from phages that infect *Erwinia*, *Escherichia*, *Klebsiella*, *Pseudomonas*, *Salmonella, Pectobacterium*, and *Vibrio*. Two Vid5 gene products showed similarity to proteins found in bacteria exclusively, as follows: hypothetical protein encoded by Vid5 ORF92 had the best match with hypothetical protein (WP_071680676.1) from *Serratia*, meanwhile the amino acid sequence of a putative PreQ_0_/PreQ_1_ transporter encoded by Vid5 ORF41 shared the highest identity with VUT family protein (WP_023656680.1) from *Erwinia piriflorinigrans* (Supplementary Table S2).

Based on homology to biologically defined proteins, 33 ORFs of Vid5 were given a putative functional annotation (Supplementary Table S2). As was observed in other viruses of the family *Siphoviridae*, the Vid5 genome appears to have a modular organization, with genes for DNA packaging, structure/morphogenesis, host lysis, replication/regulation, and nucleotide metabolism clustered together (Figure 2).

A bioinformatics analysis of the genome sequence of Vid5 allowed for the identification of twelve genes coding for proteins involved in virion structure and assembly (Supplementary Table S2). A BLASTP analysis revealed that the major capsid protein of bacteriophage Vid5 encoded by ORF6 is homologous to capsid proteins from a wide range of diverse phages, whereas HHpred yielded high probability hit to the coat protein of *Bacillus subtilis* siphophage SPP1 (4AN5_D; probability, 100%; E-value, 2.0 × 10^−47^). Three other Vid5 head-related proteins, readily identifiable by sequence homology, namely the portal protein gp03, the head-tail adaptor protein gp11, and the head completion protein gp12, show similarity to SPP1 gp6 (2JES_M; HHPred probability, 100%; E-value, 3.1 × 10^−32^), SPP1 gp15 (5A21_D; 97.04%, 8.6 × 10^−4^), and gpFII from bacteriophage lambda (2KX4.A; 96.6%, 1 × 10^−3^), respectively. Based on the BLASTP analysis, the gene product of Vid5 ORF13 shares 39% amino acid sequence identity with the tail protein gp59 of Enterobacteria phage 9g (E-value, 6 × 10^−22^), however, based on the HHsearch analysis against the ACLAME database conducted by VIRFAM, gp13 of Vid5 has been annotated as the neck protein (probability, 100%).

Three gene products of Vid5 have been assigned as tail proteins. A BLASTP analysis allowed for the identification of the major tail protein encoded by ORF15, and the tape measure protein gp18, which belongs to the Tape_meas_lam_C superfamily (cl26614). The tail completion protein Vid5 gp14, which is homologous to the hypothetical protein CPT_Seurat11 from *Escherichia* phage Seurat (49% aa ident; E value, 9 × 10^−45^), has been annotated based on a VIRFAM analysis (HHsearch probab, 96.41%). As mentioned above, only one Vid5 tail fiber/spike is visible when using TEM. Nevertheless, two putative tail fiber proteins, DUF1983 domain-containing Phage-tail_3 superfamily (cl26145) protein gp22 and the minor tail protein gp25, have been identified by the bioinformatics approaches.

FASP followed by LC-MS/MS confirmed that all of the aforementioned structural proteins, except for gp12 (head completion protein) and gp14 (tail completion protein), are present in the virion of Vid5 (Supplementary Table S3). The failure to identify gp12 and gp14 by proteomics approaches may be due to the incompatibility of these proteins with sample preparation procedures or/and because of their low abundance in virions. Notably, the proteomics analysis led to the experimental identification of gp07, which has no reliable homology to any entries in the public databases, as well as lysis regulatory protein (gp99). In addition, the putative EPS-depolymerase encoded by ORF10 was also identified, suggesting that it may be a virion-associated enzyme. The HHpred analysis suggests that the central part of Vid5 gp10 (aa 163 to 491/885) may contain lyase moiety, whereas the N terminus shows similarity to the N-terminal particle-binding domain of *Salmonella* podovirus P22 tail spike protein. Notably, the C terminus of Vid5 gp10 has no reliable homologues in databases.

The bioinformatics analysis revealed that the genes associated with Vid5 DNA packaging, replication, recombination, and repair included those coding for terminase small subunit and terminase large subunit (ORF01 and ORF02, respectively), DNA polymerase B and DNA polymerase beta subunit (ORF29 and ORF30, respectively), helicase (ORF45), putative exonuclease (ORF46), ATPase (ORF49), DNA ligase (ORF50), RNAse H (ORF51), nucleotide pyrophosphohydrolase (ORF53), putative endonuclease (ORF54), and primase (ORF61). Based on the results of the bioinformatics analysis, the TerS and TerL of Vid5 have been predicted to belong to the Terminase_2 (pfam03592) and to the Terminase_6 (pfam03237) superfamilies, respectively, and show detectable similarity to other phage-encoded TerS and TerL proteins, respectively. DNA polymerase B, encoded by the ORF29 of Vid5, has a DNA polymerase type-II B subfamily catalytic domain (cd05538) in the region between 373 and 549 amino acids (E-value, 1.39 × 10^−8^), and is followed by ORF30-encoded DNA polymerase beta subunit, which shows the highest similarity to DNA polymerase III, beta subunit from *Eubacterium rectale* (3T0P_B; HHpred probability, 100.0%; E value, 6.2 × 10^−42^). A helicase of Vid5 (ORF45) contains a conserved SSL2 superfamily domain in its N terminus (aa 1 to 387/618, E-value, 2.74 × 10^−55^), whereas the DNA ligase of Vid5 (ORF50) possesses Adenylation_kDNA_ligase_like superfamily domain (cd07896) in its N terminus (aa 3 to 189/302, E-value, 2.10 × 10^−11^) and OBF_kDNA_ligase_like superfamily domain (cd08041) in the C terminus (aa 231 to 296/302, E-value, 5.89 × 10^−10^).

Phage-host interaction and lysis proteins, identified in the genome of Vid5, included endolysin, (DUF3380 superfamily (cl13324)), Phage_holin_3_3 domain-containing holin (cl24062), and a putative phage lysis regulatory protein (DUF2570 superfamily, cl24005), encoded by ORF97, ORF98, and ORF99, respectively. In addition, a gene cluster encoding enzymes possibly involved in the biosynthesis of 7-deazaguanine derivatives has been detected. Notably, none of the predicted Vid5 proteins showed sequence homology with integration-related proteins, antibiotic resistance determinants, or virulence factors.

### 3.3. Phylogenetic Analysis

**Figure 3:**
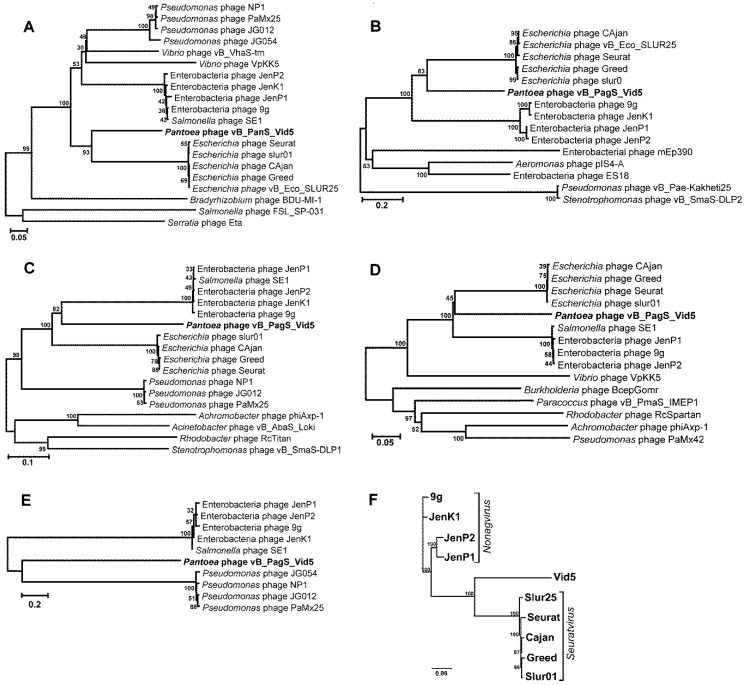
Neighbor-joining tree analysis based on the alignment of the amino acid sequences of: (**A**) major capsid protein, (**B**) tape measure protein, (**C**) polymerase B, (**D**) terminase large subunit, and (**E**) glutamine amidotransferase/7-cyano-7-deazaguanine synthase (gat-queC). Phylogenetic analysis was conducted using MEGA version 5. The percentage of replicate trees in which the associated taxa clustered together in the bootstrap test is shown next to the branches. (**F**) Neighbor-joining tree based on the alignment of Vid5 as well as *Nonagvirus* and *Seuratvirus* phage genome sequences available in NCBI GenBank. The tree was constructed using Geneious v5.5.6; the numbers at the nodes indicate the bootstrap probabilities.

The VIRFAM analysis, a “head-neck-tail”-based classification method proposed by Lopes and coauthors [28], classified Vid5 as a siphovirus of Type 1 (Cluster 5) (Supplementary Figure S2), suggesting that this phage adopts the structural organization of the *Siphoviridae* phage SPP1 neck. Notably, cluster five is composed strictly of siphophages, of which all but two, *Streptomyces* phages phi-C31 and phi-BT1, infect *Proteobacteria* [28]. However, a VIRFAM analysis revealed no phylogenetic relationship between Vid5 and its closest relatives identified by bioinformatics approaches. Thus, the comparison of individual genes often used for the analysis of the evolutionary relationships among bacteriophages [35] was carried out. Four phylogenetic trees based on the alignment of the Vid5 polymerase, terminase large subunit, major capsid, and tape measure protein sequences with those returned by BLASTP homology searches were constructed (Figure 3).

As seen in Figure 3, in all four of the phylogenetic trees bacteriophage Vid5 represents a distinct branch that seems to occupy a somewhat intermediate position between Enterobacteria siphophages, which belong to the *Nonagvirus* genus (https://talk.ictvonline.org/ICTV/proposals/2015.051a-dB.A.v2.Nonagvirus.pdf), and those that represent the genus *Seuratvirus* (https://talk.ictvonline.org/ICTV/proposals/2015.053a-dB.A.v2.Seuratvirus.pdf). According to the 2017 ICTV taxonomy release (https://talk.ictvonline.org/taxonomy/), the genus *Nonagvirus* includes four Enterobacteria viruses, 9g [36], JenK1, JenP1 and JenP2 [37], whereas phages Seurat [38] and Cajan [20] have been assigned to the genus *Seuratvirus*. Last year, 14 novel phages potentially belonging to the genus *Seuratvirus* were described by Sazinas and coauthors [39]. However, the complete genome sequences of only three of those viruses, namely *Escherichia* phage slur01 [40], Greed [41], and vB_Eco_SLUR25 [39], have been deposited in the NCBI database to date.

To obtain a more detailed picture of the phylogenetic relationships between the viruses classified within the genera *Nonagvirus* and *Seuratvirus* and phage Vid5, the genome sequences of nine Enterobacteria phages were compared to that of Vid5 using mVISTA. A comparative whole-genome sequence alignment revealed that the genome of Vid5 shared several regions of nucleotide sequence similarity with the genomes of all of the phages analyzed. In the case of Vid5, the aforementioned regions covered the virion morphogenesis as well as DNA metabolism and modification genes (Supplementary Figure S3). Nevertheless, the overall nucleotide sequence similarity between Vid5 and the type phages of *Nonagvirus* and *Seuratvirus* was quite low, and ranged from 33.08% (Vid5 vs. 9g) to 34.23% (Vid5 vs. Seurat). Also, as seen in Figure 3F, Vid5 represents a distinct branch on the neighbor-joining tree, based on the whole-genome sequence alignment of Vid5 and nine phage genomes analyzed.

According to the Bacterial and Archaeal Viruses Subcommittee (BAVS) of the ICTV, a genus is a cohesive group of viruses sharing a high degree (>50%) of nucleotide sequence similarity [42]. Following this, and based on the results of the comparative genome sequence analysis conducted during this study, bacteriophage Vid5 cannot be assigned to any genus currently recognized by ICTV and likely represents a new genus within the family *Siphoviridae*.

### 3.4. The Gene Cluster Potentially Involved in the Biosynthesis of 7-Deazaguanine Derivatives

**Figure 4:**
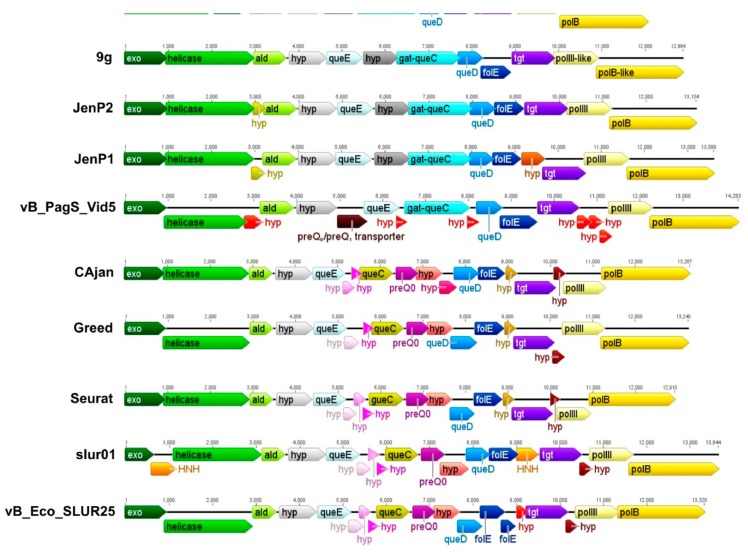
Comparison of gene clusters involved in the biosynthesis of 7-deazaguanines in Vid5 and related phages. *Exo*—exonuclease; *ald*—aldolase; *hyp*—hypothetical protein; *preQ_0_/preQ_1_*—7-cyano-7-deazaguanine/7-aminomethyl-7-deazaguanine transporter; *queE*—7-carboxy-7-deazaguanine synthase; *queC*—7-cyano-7-deazaguanine synthase; *gat-queC*—glutamine amidotransferase/7-cyano-7-deazaguanine synthase; *queD*—6-carboxytetrahydropterin synthase; *folE*—GTP cyclohydrolase I; *tgt*—tRNA-guanine transglycosylase; *HNH*—homing endonuclease H; *polIII*—DNA polymerase β-subunit; *polB*—DNA polymerase B. Same color indicates ORFs that encode homologous proteins, with the exception of the color red, which indicates ORFs that encode unique proteins with no reliable identity to database entries.

As mentioned previously, a bioinformatics analysis of Vid5 genome revealed the presence of the gene cluster *que*-*tgt* possibly involved in the biosynthesis of 7-deazaguanine derivatives. Based on a comparative sequence analysis, the gene cluster *que*-*tgt* of Vid5 is similar to that of *Escherichia coli* phage 9g, the prototype of the genus *Nonagvirus* (Figure 4). In the case of bacteriophage 9g, up to 27% of the G bases in genomic DNA are converted to archeosine (G^+^) [43]. According to the literature, the synthesis of G^+^ is a complex pathway requiring the 7-cyano-7-deazaguanine intermediate (preQ_0_) [44]. As observed with 9g, the genome of Vid5 contains three out of four genes essential for the biosynthesis of preQ_0_, namely GTP cyclohydrolase I (*folE*), 6-carboxy-5,6,7,8-tetrahydropterin synthase (*queD*), and 7-carboxy-7-deazaguanine synthase (*queE*) (Supplementary Table S2). Both phages 9g and Vid5 also code for a Tgt-like protein and Gat-QueC*.* Tgt-like protein is thought to be involved in inserting 7-deazaguanine derivatives in DNA, whereas Gat-QueC is a homolog of QueC fused to a glutamine amidotransferase class-II domain (GATase), and has been suggested to catalyze the conversion of preQ_0_ to G+ [44]. Following these observations, we suggest that G+ is likely present in the DNA of Vid5.

Although further experiments are needed to confirm the aforementioned assumption, according to Tsai and colleagues, the presence of dG+ in the 9g genome renders the DNA resistant to many of the Type II REases [45]. Based on this, the susceptibility of phage Vid5 genomic DNA to several REases was compared to that reported for 9g. The results of restriction digestion analysis (Supplementary Figure S4) revealed that the genomic DNA of phage Vid5 was completely resistant to Eco321 (GAT↓ATC), BamHI (G↓GATCC), MboI (↓GATC), EcoRII (↓CCWGG), and Bsu151 (AT↓CGAT). Two REases with AT rich recognition sequences, NdeI (CA↓TATG) and MfeI (C↓AATTG), partially digested phage Vid5 gDNA, meanwhile DraI (only A/T in its recognition sequence TTT↓AAA) was capable of digesting Vid5 DNA to completion. Similar to what was observed for 9g, despite the presence of guanines in their recognition sequences, three REases, namely RsaI (GT↓AC), HhaI (GCG↓C), and Csp61 (G↓TAC), were also able to digest Vid5 DNA to completion. Overall, as the susceptibility of phage Vid5 genomic DNA to all TypeII REases listed above is comparable to that observed for 9g, it is likely that both phages contain similar DNA modifications.

## 4. Discussion

There are only two genome sequences of bacteriophages annotated as *Pantoea* phages currently available in Genbank. Both of these *Pantoea* phages, namely bacteriophages LIMElight and LIMEzero, are classified within the *Autographivirinae* subfamily of the *Podoviridae*. However, a number of broad-host range *Erwinia* or *Serratia* phages have been reported to be able to infect bacteria from the genus *Pantoea* [4,5,6,7,8,9,16]. To our knowledge, all of these bacteriophages, with the exception of siphoviruses PhiEaH1 [12], PhiEaH2 [13], KEY [14], and phiOT8 [16], have been assigned to the families *Myoviridae* or *Podoviridae*. However, the genome sequences of only two of the aforementioned viruses, *Erwinia* phages PhiEaH1 and PhiEaH2, have been published to date, but there is no publicly available information on the host range of these bacteriophages. Therefore, it is unclear whether these phages are capable of infecting *Pantoea*. In addition, with the genome size of 61,437 bp, phage Vid5 rather differs from phages PhiEaH1 and PhiEaH2, which possess genomes of 218,339 bp and 243,050 bp, respectively. In contrast to PhiEaH1 and PhiEaH2, *Erwinia* phage KEY and *Serratia* phage phiOT8 have been shown to infect *Pantoea* sp.*,* but no complete genome sequences of these phages have been published so far. Hence, based on the data presented in the literature to date, we state that bacteriophage Vid5 is the first *Pantoea*-infecting siphovirus with completely sequenced and publicly available genome.

A whole-genome sequence comparison using Megablast revealed that the Vid5 genome produces no significant matches to any GenBank entries. However, when compared using the BlastN algorithm, Vid5 shares 65% identity over 14% of its genome with that of Enterobacteria phage 9g. Bacteriophage 9g is a siphovirus of the genus *Nonagvirus*, and has been shown to modify its DNA with G^+^. As discussed in the previous section, bacteriophage Vid5 has homologues of archeosine biosynthesis genes as well, and the gene cluster responsible for this potential modification in Vid5 is rather similar in the overall structure and organization to that of 9g, suggesting that the DNA of Vid5 may also contain dG^+^. Moreover, the restriction digestion analysis suggests that the genomes of both Vid5 and 9g contain similar DNA modifications.

7-deazaguanine derivatives have long been thought to exist exclusively in tRNA, however, recent studies indicate that certain phages may incorporate 7-aminomethyl-7-deazaguanine (preQ_1_), 7-cyano-7-deazaguanine (preQ_0_), 7-amido-7-deazaguanine (ADG), or 7-formamidino-7-deazaguanosine (archaeosine, G^+^) into their DNA [39,44,45]. It has been suggested that phages may incorporate 7-deazaguanine derivatives into DNA so as to protect their genomes from the restriction enzymes of the host [36,39,43,45]. Whatever the case, out of all of the genomes of bacterial viruses that are currently available in pVOG and NCBI databases, only 32 contain all four genes (*fol*E, *que*D, *que*E, and *que*C) essential for the biosynthesis of preQ_0_, the precursor for all 7-deazapurines [39]. Of these phages, 18 are classified within the *Seuratvirus* genus and have been suggested to modify their DNA with 7-deazapurines other than G^+^. As these phages contain no *que*A, *que*F, and *que*G homologs, they may be predicted to insert preQ_0_ or ADG into their DNA [44].

Bacteriophage Vid5 has no homologue to QueC, the 7-Cyano-7-deazaguanine synthase that catalyzes the formation of preQ_0_ from 5-carboxydeazaguanine. However, as mentioned in the previous section, the DNA of Vid5 codes for a Gat-QueC, indicative of the presence of G^+^ in its DNA. Based on our results, the homologues to glutamine amidotransferase/7-cyano-7-deazaguanine synthase Gat-QueC may be found only in the members of the genus *Nonagvirus* and four *Pseudomonas* phages, namely NP1, PaMx25, JG012, and JG054 (Figure 3E). Notably, all *Nonagviruses* contain no *que*C homologues, yet they have been suggested to possess G^+^ in their DNA. All of this raises the question, how do these viruses and Vid5 obtain the necessary G^+^ precursor preQ_0_? One of the most obvious solutions would be to use the host-encoded QueC. Also, a possibility exists that one of the hypothetical proteins encoded within the gene cluster *que*-*tgt* of these phages acts in an analogous manner to QueC. However, as seen in Figure 4, unlike other phages that harbor the genes for the biosynthesis of 7-deazaguanine derivatives, Vid5 also codes for a putative 7-cyano-7-deazaguanine/7-aminomethyl-7-deazaguanine (preQ_0_/preQ_1_) transporter that is similar to the preQ_0_/preQ_1_ transporters from various bacteria, yet has no homologs in bacteriophages. The Vid5 preQ_0_/preQ_1_ transporter has been predicted to belong to the Vut_1 superfamily, which includes the COG1738 family proteins. Last year, the COG1738 family protein YhhQ from *Escherichia coli* was proved to be involved in the preQ_0_/preQ_1_ salvage [46]. Notably, six transmembrane segments, characteristic of transporters, have been predicted in Vid5 PreQ_0_/PreQ_1_ by bioinformatics approaches. Therefore, it may be hypothesized that, in the case of bacteriophage Vid5, the preQ_0_ precursor may be salvaged. According to the literature, preQ_0_ is available in natural environments as an intermediate of the Q or G^+^ pathways, as well as a secondary metabolite or secondary metabolite precursor [44,47]. As shown in the Results section, bacteriophage Vid5 is a low-temperature virus capable of propagating even at 4 °C. Hence, it is possible that at higher temperatures, this phage either takes advantage of the host-encoded QueC, or uses its own as of yet unidentified QueC analogue. In contrast, at a low temperature, because of a reduced rate of protein synthesis, Vid5 employs salvaged preQ_0_ for the synthesis of G^+^.

In conclusion, we have shown that *Pantoea* sp. infecting bacteriophage Vid5 is a low-temperature siphovirus possessing the archaeosine biosynthesis genes, including *pre*Q_0_/*pre*Q_1_, which has never been detected in bacteriophage genomes to date. In addition, our results indicate that Vid5 is substantially distinct from all of the previously described phages, and may be considered as a representative of a novel genus within the family *Siphoviridae*. Also, according to the literature, only a limited number of low-temperature enterobacteria phages have been described to date, and little is known about the molecular mechanisms underlying the cold-adaptation of bacterial viruses [48,49,50], such as bacteriophage Vid5, which is adjusted to replicate at lower temperatures, and may be a suitable model object for studying this phenomenon.

## Supplementary Materials

The following are available online at http://www.mdpi.com/1999-4915/10/11/583/s1, Table S1: Bacterial strains used in this study to determine the host range of phage Vid5; Table S2: Vid5 ORFs with homologues in other viruses or cellular organisms; Table S3: Structural Vid5 proteins identified by MS; Figure S1: Effect of temperature on the efficiency of plating of phage Vid5; Figure S2: VIRFAM-generated clustering of Vid5 with the phages sharing the most similar head-neck-tail module, Figure S3: A whole-genome alignment generated using mVISTA, Figure S4: Restriction digestion patterns of phage Vid5 genomic DNA.
