# Supplementary material for: Pantoea Bacteriophage vB_PagS_Vid5: A Low-Temperature Siphovirus That Harbors a Cluster of Genes Involved in the Biosynthesis of Archaeosine
Source: Viruses. 2018 Oct 25;10(11):583. doi: 10.3390/v10110583 (PMC6266253; doi:10.3390/v10110583)
Supplement: Supplementary file 1 [file viruses-10-00583-s001.pdf]

Supplementary Table S1. Bacterial strains used in this study to determine the host range of phage Vid5.

Strain	Relevant characteristics	Source or reference
Acinetobacter baumannii #46		Prof. E. Suziedeliene
Acinetobacter baumanni 13#23		Prof. E. Suziedeliene
Arthrobacter koreensis DSM 16760	Type strain	DSMZ
Arthrobacter histidinivirans DSM 20115	Type strain	DSMZ
Arthrobacter ureafaciens DSM 20126	Type strain	DSMZ
Citrobacter freundii		Prof. E. Suziedeliene
Enterobacter cloacea		Prof. E. Suziedeliene
Erwinia carotovora 8982		Prof. E. Suziedeliene
Erwinia carotovora 961–63		Prof. E. Suziedeliene
Escherichia coli B40	*sup*D	Dr. L. W. Black
Escherichia coli B^E	sup^0	Dr. L. W. Black
Escherichia coli BL21	F^- *dcm ompT hsdS* (rB^-mB^-) *gal*	Novagen
Escherichia coli BW25113	[Δ(*araD–araB*)*567* Δ(*rhaD–rhaB*)*568 ΔlacZ4787* (::rrnB-3) *hsdR514 rph-1*]	[51]
Escherichia coli DH10β	F^- *endA1 recA1 galE15 galK16 nupG rpsL* Δ*lacX74* Φ80*lacZ*ΔM15 *araD139* Δ(*ara,leu*)7697 *mcrA* Δ(*mrr-hsdRMS-mcrBC*) λ^-	Invitrogen
Escherichia coli MG1655	F^- lambda$^-$ *ilvG*$^-$ *rfb*-50 *rph*-1	Prof. E. Suziedeliene
Escherichia coli MH1	*araD139 ΔlacX74 galU galK hsr hsm rpsL*	Dr. K. N. Kreuzer
Klebsiella sp. KV-3	Veterinary isolate, Amp^r, Str^r, Tet^r, Kan^s, Gm^s, Nc^s,$Cl^{r/s}$	[21]
Klebsiella pneumoniae 279		Prof. E. Suziedeliene
Pantea agglomerans ARC	environmental isolate	this study
Pantea agglomerans AUR	environmental isolate	this study
Pantea agglomerans BSL	environmental isolate	this study
Pantea agglomerans DDM	environmental isolate	this study
***Pantea agglomerans* MMG**	environmental isolate	this study
Pantea agglomerans SER	environmental isolate	this study
Pseudomonas aeruginosa PAO1		Prof. E. Suziedeliene
Salmonella enterica ser. Typhimurium 292		Prof. E. Suziedeliene

Vid5-sensitive strain is marked in bold.

Supplementary Table S2. Vid5 ORFs with homologues in other viruses or cellular organisms.

Vid5 ORF (position)	Predicted function (protein length aa)	Significant match (protein length aa)	Identity aa %/ similarity aa% (length of the overlapping segment)	E value
ORF01	terminase small subunit	YP_009151945.1 TerS	48/68 (172)	1e-51

(1..546)	(181)	*Escherichia* phage Seurat (185)		
ORF02 (533..2119)	terminase large subunit (528)	YP_009216940.1 terminase large subunit Enterobacteria phage JenP2 (532)	69/82 (529)	0.0
ORF03 (2131..3657)	portal protein (508)	SOE45329.1 putative portal protein *Escherichia* phage vB_Eco_SLUR25 (498)	57/72 (485)	0.0
ORF04 (3679..4605)	hypothetical protein (308)	ARB11150.1 hypothetical protein JG054_00005 *Pseudomonas* phage JG054 (272)	51/67 (258)	4e-71
ORF06 (4741..5832)	major capsid protein (363)	ANY29798.1 capsid protein *Escherichia* phage Greed (353)	60/73 (341)	1e-136
ORF08 (6022..6921)	hypothetical protein (299)	ARM70072.1 hypothetical protein *Salmonella* phage SE1 (223)	49/70 (71)	2e-14
ORF10 (7122..9779)	EPS-depolymerase (885)	YP_009147633.1 EPS-depolymerase *Erwinia* phage phiEa2809 (939)	28/42 (553)	3e-50
ORF11 (9788..10297)	head-tail adaptor (169)	YP_009196802.1 structural protein *Escherichia* phage CAjan (168)	43/57 (157)	1e-34
ORF12 (10297..10674)	head completion protein (125)	YP_009216948.1 hypothetical protein Enterobacteria phage JenP2 (124)	34/51 (123)	4e-14
ORF13 (10671..11075)	neck protein (134)	YP_009201593.1 unnamed protein product *Escherichia* phage slur01 (135)	43/52 (129)	3e-26
ORF14 (11072..11533)	tail completion protein (153)	YP_009151955.1 hypothetical protein CPT_Seurat11 *Escherichia* phage Seurat (149)	49/62 (150)	6e-43
ORF15 (11548..12480)	major tail protein (310)	SOE45367.1 tail subunit *Escherichia* phage vB_Eco_SLUR25 (307)	66/79 (307)	3e-147
ORF16 (12497..12937)	hypothetical protein/tape measure chaperone (146)	YP_009216952.1 hypothetical protein Enterobacteria phage JenP2 (142)	46/62 (140)	1e-34
ORF17 (12979..13314)	hypothetical protein/tape measure chaperone (111)	YP_009219985.1 hypothetical protein Enterobacteria phage JenP1 (112)	49/67 (102)	3e-29
ORF18 (13314..16577)	tape measure protein (1087)	ANY29808.1 tail length tape-measure protein 1 *Escherichia* phage Greed (1038)	42/58 (1105)	0.0
ORF19 (16580..17170)	hypothetical protein (196)	ANY29809.1 hypothetical protein *Escherichia* phage Greed (194)	52/70 (193)	5e-69
ORF20 (17170..17757)	hypothetical protein (195)	YP_009032389.1 hypothetical protein 9g_00066 Enterobacteria phage 9g (194)	52/70 (195)	7e-66
ORF21 (17765..18166)	hypothetical protein (219)	ARM70058.1 hypothetical protein *Salmonella* phage SE1 (144)	47/60 (138)	3e-30
ORF22 (18168..20891)	central tail fiber protein (907)	ANY29812.1 tail fiber protein *Escherichia* phage Greed (1040)	53/67 (889)	0.0
ORF23 (20891..21871)	hypothetical protein (326)	ATS94092.1 hypothetical protein P13BB106kb_p108 *Pectobacterium* phage DU_PP_V (222)	27/42 (143)	8e-07
ORF25 (22230..23465)	minor tail protein (411)	ARB10877.1 minor tail protein *Salmonella* phage 29485 (432)	33/51 (432)	1e-54
ORF29 (complement, 24372..26402)	DNA polymerase B (676)	YP_009151974.1 DNA polymerase *Escherichia* phage Seurat (661)	53/68 (681)	0.0
ORF30 (complement, 26399..27361)	DNA polymerase beta subunit (320)	YP_009285844.1putative replicative clamp *Pseudomonas* phage NP1 (317)	53/68 (320)	9e-119
ORF34 (complement, 28094..29014)	queuosine tRNA ribosyltransferase (tgt) (306)	ARB11177.1 putative queuosine tRNA ribosyltransferase *Pseudomonas* phage JG054 (312)	77/89 (312)	0.0
ORF35	GTP cyclohydrolase type	ANO57492.1 type 1 GTP cyclohydrolase I	77/87 (163)	6e-89

(complement, 29083..29898)	I (folE)(271)	*Vibrio* phage vB_VhaS-tm (199)		
ORF36 (complement, 29895..30440)	6-carboxytetrahydropterin synthase (queD) (181)	YP_009220005.1 6-carboxytetrahydropterin synthase Enterobacteria phage JenP1 (171)	70/76 (179)	2e-79
ORF38 (complement, 30649..32112)	glutamine amidotransferase class-II (GATase)/ 7-cyano-7-deazaguanine synthase (gat-queC) (487)	ARM70135.1 glutamine amidotransferases class-II (GATase)/7-cyano-7-deazaguanine synthase *Salmonella* phage SE1 (471)	56/68 (495)	2e-179
ORF40 (complement, 32302..33060)	queuosine biosynthesis protein queE (252)	ANY29839.1 queuosine biosynthesis QueE radical SAM *Escherichia* phage Greed (235)	54/69 (252)	3e-93
ORF41 (complement, 33050..33688)	$preQ_0/preQ_1$ transporter (212)	WP_023656680.1 VUT family protein *Erwinia piriflorinigrans* (221)	55/75 (196)	6e-76
ORF42 (complement, 33753..34640)	transcriptional activator (295)	YP_009216976.1 hypothetical protein Enterobacteria phage JenP2 (274)	45/63 (226)	2e-48
ORF43 (complement, 34755..35486)	hypothetical protein (243)	YP_009032333.1 hypothetical protein 9g_00010 Enterobacteria phage 9g (240)	41/59 (243)	4e-53
ORF45 (complement, 35863..37719)	helicase (618)	YP_009196842.1 helicase-like protein *Escherichia* phage Cajan (658)	58/71 (622)	0.0
ORF46 (complement, 37719..38654)	putative exonuclease (311)	ARM70128.1 hypothetical protein *Salmonella* phage SE1 (316)	58/70 (311)	1e-127
ORF48 (complement, 38995..39984)	hypothetical protein (329)	YP_009285858.1 hypothetical protein *Pseudomonas* phage NP1 (181)	44/61 (167)	1e-37
ORF49 (complement, 39989..40708)	ATPase (239)	YP_009285859.1 ATPase *Pseudomonas* phage NP1 (241)	62/76 (240)	4e-103
ORF50 (complement, 40771..41679)	DNA ligase (302)	YP_009196846.1 putative ATP-dependent DNA ligase *Escherichia* phage Cajan (304)	56/72 (300)	4e-124
ORF51 (complement, 41681..42346)	RNAse H (221)	YP_009219325.1 RNAse H Enterobacteria phage JenK1 (196)	42/59 (149)	1e-33
ORF52 (complement, 42346..42762)	hypothetical protein (138)	YP_009216985.1 hypothetical protein Enterobacteria phage JenP2 (141)	44/60 (140)	2e-28
ORF53 (complement, 42829..43656)	nucleotide pyrophosphohydrolase (275)	ALH23773.1 putative nucleotide pyrophosphohydrolase *Pseudomonas* phage PaMx25 (130)	44/66 (111)	2e-21
ORF54 (complement, 43666..44121)	putative endonuclease (151)	ARM70119.1 hypothetical protein *Salmonella* phage SE1 (153)	63/75 (150)	4e-65
ORF61 (complement, 45645..47987)	primase (780)	YP_009201557.1 primase *Escherichia* phage slur01 (780)	58/73 (782)	0.0
ORF62 (complement,	hypothetical protein (77)	YP_009152005.1 hypothetical protein CPT_Seurat61	42/61 (57)	6e-06

47998..48231)		*Escherichia* phage Seurat (67)		
ORF76 (complement, 52121..52498)	hypothetical protein (125)	YP_008550179.1 hypothetical protein AV954_gp66 *Escherichia* virus SSL2009a (57)	60/71 (52)	6e-11
ORF86 (56396..56587)	hypothetical protein (83)	AQT28529.1 hypothetical protein YOLOSWAG_44 *Erwinia* phage vB_EamM_Yoloswag (101)	55/80 (51)	2e-11
ORF92 (57829..58074)	hypothetical protein (85)	WP_071680676.1 hypothetical protein *Serratia fonticola* (74)	53/59 (74)	1e-15
ORF93 (58071..58538)	hypothetical protein (155)	ANY29786.1 hypothetical protein *Escherichia* phage Greed (140)	35/48 (150)	6e-17
ORF94 (58535..58903)	hypothetical protein (122)	APU92831.1 hypothetical protein CPTSasha_75 *Salmonella* phage vB_SenS_Sasha (131)	45/54 (130)	2e-29
ORF95 (58900..59379)	hypothetical protein (159)	YP_009220052.1 hypothetical protein Enterobacteria phage JenP1 (164)	32/48 (155)	5e-10
ORF97 (59731..60333)	endolysin (200)	ASV45028.1 endolysin *Klebsiella* phage SopranoGao (190)	51/67 (198)	2e-63
ORF98 (60363..60758)	holin (131)	YP_009285813.1 hypothetical protein *Pseudomonas* phage NP1 (121)	38/61 (121)	8e-21
ORF99 (60739..61299)	lysis regulatory protein (186)	YP_009152032.1 hypothetical protein CPT_Seurat88 *Escherichia* phage Seurat (158)	36/55 (144)	7e-17

Supplementary Table S3. Structural Vid5 proteins identified by MS.

Gene	Putative function	MW (KDa)	Peptide count	Sequence coverage (%)
ORF18	tape measure protein	115.063	69	56.95
ORF22	central tail fiber protein	98.324	7	10.25
ORF10	EPS depolymerase	95.823	76	65.31
ORF03	portal protein	54.818	48	67.0
ORF25	minor tail protein	44.464	13	27.98
ORF06	major capsid protein	39.892	34	53.17
ORF15	major tail protein	33.329	16	47.10
ORF99	lysis regulatory protein	20.453	12	73.66
ORF11	head-tail adaptor	18.734	2	17.16
ORF13	neck protein	14.569	3	27.61
ORF07	hypothetical protein	4.780	2	57.5

Vid5 specific ORF with no reliable identity to database entries is underlined

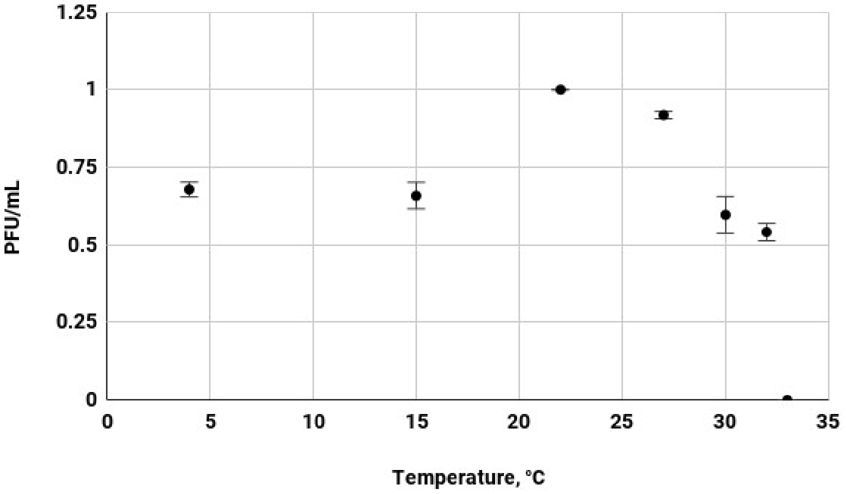

Supplementary Figure S1. Effect of temperature on the efficiency of plating of phage Vid5. Each point represents the mean of three individual experiments.

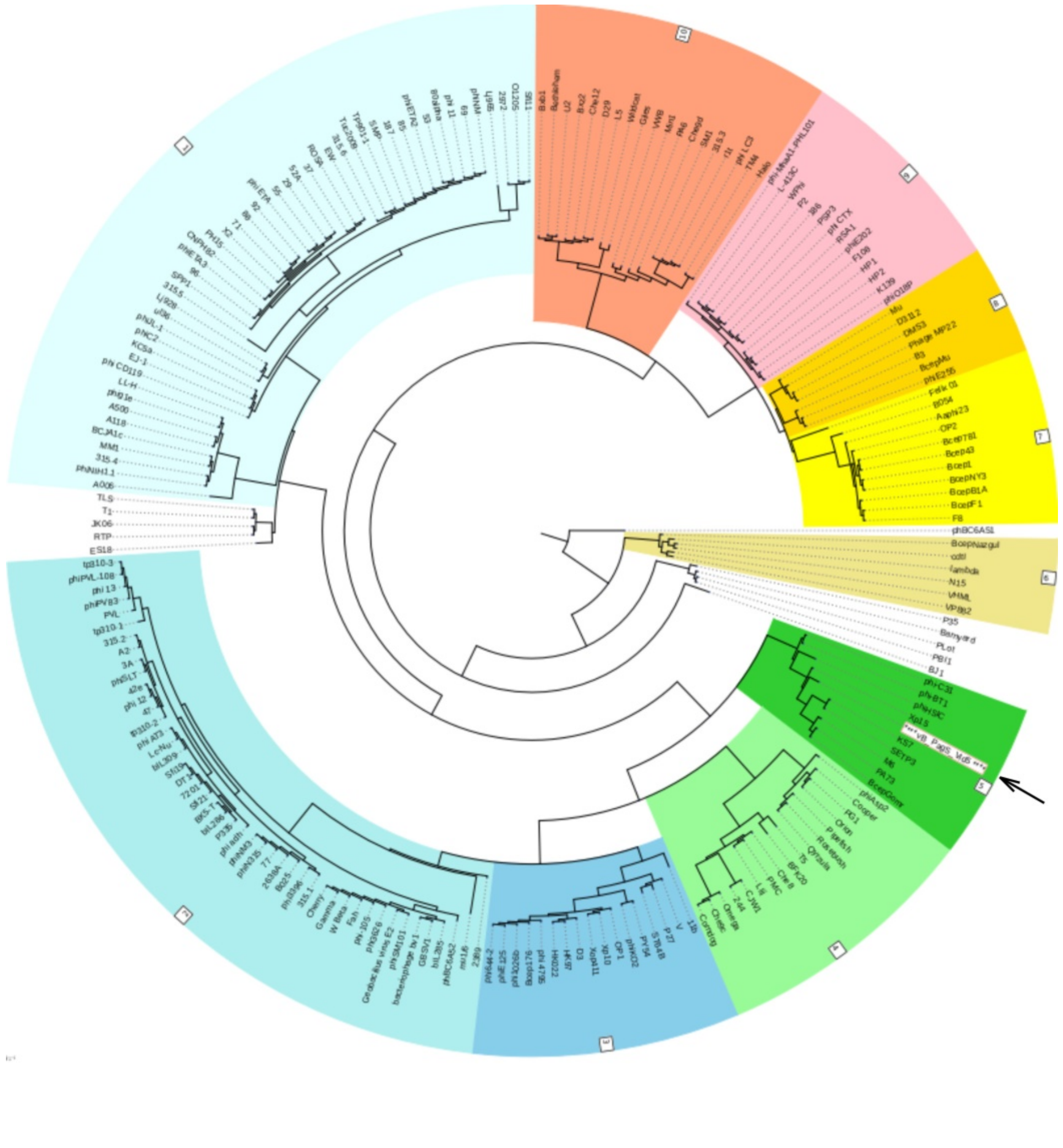

Supplementary Figure S2. VIRFAM-generated clustering of Vid5 with the phages sharing the most similar head-neck-tail module. Different Type 1 phage clusters are highlighted by different background colours. Vid5 is indicated by the black arrow.

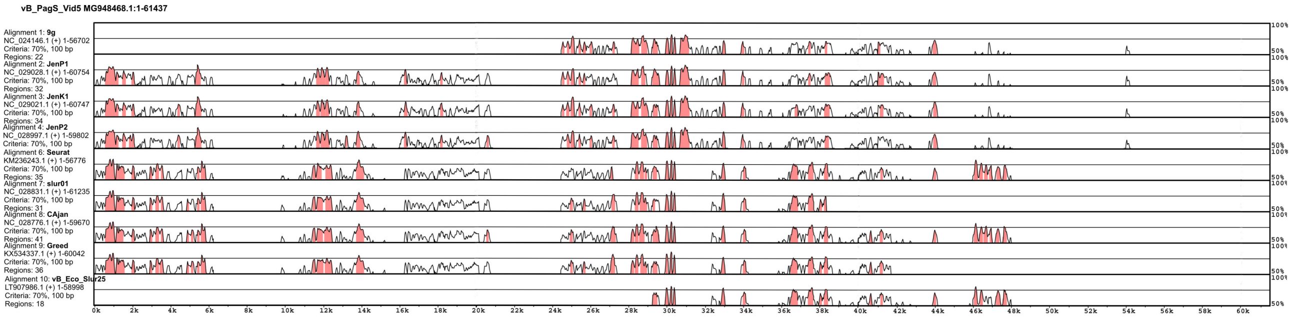

Supplementary Figure S3. A whole-genome alignment generated using mVISTA. Vid5 genome sequence aligned with the genome sequences of phages from the genera *Nonagviruses* and *Seuratviruses*. Red blocks represent aligned regions, and similarity is indicated by the height of bars.

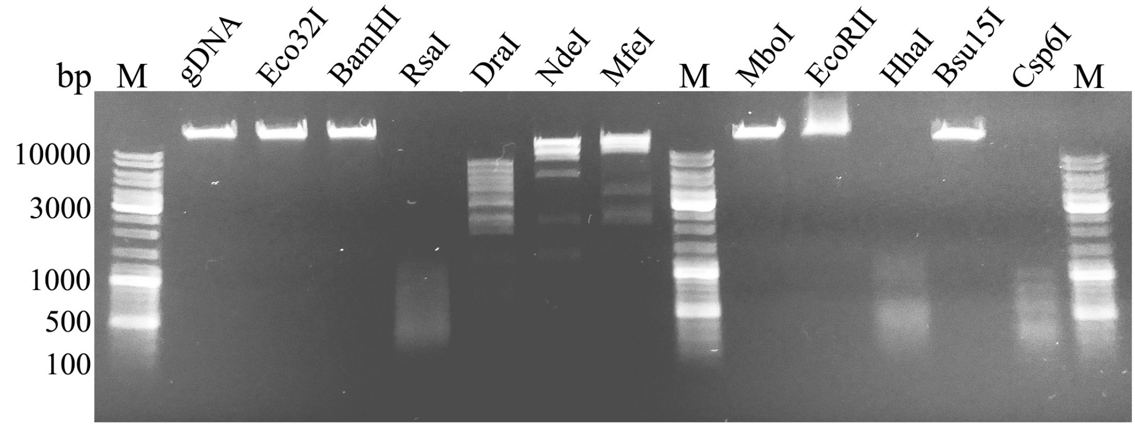

Supplementary Figure S4. Restriction digestion patterns of phage Vid5 genomic DNA. *M*, GeneRuler™ DNA Ladder Mix (Thermo Fisher Scientific); gDNA, undigested genomic DNA of Vid5.

References

51. Hyman, P.; Abedon, S.T. Bacteriophage host range and bacterial resistance. *Adv. Appl. Microbiol.* **2010**, *70*, 217–248, doi:10.1016/S0065-2164(10)70007-1.
